# The Effects of Perchlorates on the Permafrost Methanogens: Implication for Autotrophic Life on Mars

**DOI:** 10.3390/microorganisms3030518

**Published:** 2015-09-09

**Authors:** Viktoria Shcherbakova, Viktoria Oshurkova, Yoshitaka Yoshimura

**Affiliations:** 1 Skryabin Institute of Biochemistry and Physiology of Microorganisms, Russian Academy of Sciences, Prospect Nauki 5, Pushchino, Moscow, 142290, Russia; E-Mail: vicyle4ka@gmail.com; 2 Japan Aerospace Exploration Agency, Institute of Space and Astronautical Science (ISAS), 3-1-1 Yoshinodai, Chuo-ku, Sagamihara, Kanagawa, 252-5210, Japan; E-Mail: ystk@agr.tamagawa.ac.jp; 3 College of Agriculture, Tamagawa University, 6-1-1 Tamagawagakuen, Machida, Tokyo, 194-8610, Japan

**Keywords:** permafrost, Mars, methanogenic archaea, perchlorates

## Abstract

The terrestrial permafrost represents a range of possible cryogenic extraterrestrial ecosystems on Earth-like planets without obvious surface ice, such as Mars. The autotrophic and chemolithotrophic psychrotolerant methanogens are more likely than aerobes to function as a model for life forms that may exist in frozen subsurface environments on Mars, which has no free oxygen, inaccessible organic matter, and extremely low amounts of unfrozen water. Our research on the genesis of methane, its content and distribution in permafrost horizons of different ages and origin demonstrated the presence of methane in permanently frozen fine-grained sediments. Earlier, we isolated and described four strains of methanogenic archaea of *Methanobacterium* and *Methanosarcina* genera from samples of Pliocene and Holocene permafrost from Eastern Siberia. In this paper we study the effect of sodium and magnesium perchlorates on growth of permafrost and nonpermafrost methanogens, and present evidence that permafrost hydogenotrophic methanogens are more resistant to the chaotropic agent found in Martian soil. In this paper we study the effect of sodium and magnesium perchlorates on the growth of permafrost and nonpermafrost methanogens, and present evidence that permafrost hydogenotrophic methanogens are more resistant to the chaotropic agent found in Martian soil. Furthermore, as shown in the studies strain M2^T^ *M. arcticum*, probably can use perchlorate anion as an electron acceptor in anaerobic methane oxidation. Earth’s subzero subsurface environments are the best approximation of environments on Mars, which is most likely to harbor methanogens; thus, a biochemical understanding of these pathways is expected to provide a basis for designing experiments to detect autotrophic methane-producing life forms on Mars.

## 1. Introduction

In 1974 Cameron and Morelli were the first to advance the idea for solving the exobiology problems by using the terrestrial permafrost model [1]. Permafrost is a stable and balanced environment, which maintains life incomparably longer than any other known habitats. It does not depend on the composition of the ground and acts as a physical and biogeochemical barrier that limits infiltration of both surface water and external environmental factors [2,3].Viable microorganisms have been isolated from permanently frozen sediments at lowest temperatures in the Arctic (−17 °C) and Antarctica (−27 °C) [4,5,6], down to 400 m depth in the Mackenzie Delta [4], and up to 4700 m elevation in the Tibetan Plateau [7]. The age of the isolates indicates the longevity of the permanently frozen state of the sediments, and dates back from a few thousand to 2–3 million years in eastern Arctic, and to 5–8 million years in Antarctica. This is why permafrost on Earth has been considered a terrestrial analogue for possible extraterrestrial ecosystems within frozen subsurfaces, such as is assumed for Mars.

Mars presents hostile environments for mesophilic terrestrial life [8,9,10], but it seems to be capable of supporting liquid water, which is generally regarded as a prerequisite for life. Results from the Neutron Spectrometer on Mars Odyssey indicate the presence of extensive deposits of water ice [11,12,13,14]. As it is known by now, the top 20–50 cm of Martian surface is friable dry frozen ground, and the subsequent 1 m that was investigated is permafrost [10]. Methane was discovered in the Martian atmosphere in 2003, and, to date, several separate groups have reported its detection [15,16,17,18]. Because of the photochemical dissociation of tropospheric methane, methane has a lifetime of only several hundred years in the troposphere. Hitchcock and Lovelace [19] were the first to point out that, because of its short lifetime, the presence of atmospheric methane indicates its constant replenishment. This replenishment could originate either from biological metabolism or from non-biological processes (volcanoes, *etc.*).

Thus, the anaerobic chemolithotrophic psychrotolerant–methanogenic microorganisms with their mechanisms to assimilate CO_2_ and other inorganic compounds are more likely than aerobes to function as a model for life forms that may exist in frozen subsurface environments on Mars, which has no free oxygen, inaccessible organic matter, and extremely low amounts of unfrozen water. Earlier, we isolated and described four strains of psychrotolerant methanogens from the samples of Pliocene and Holocene permafrost from Eastern Siberia [20,21,22]. Methane-producing *Archaea* obtain energy in converting a limited number of substrates [23,24]. The main substrates are CO_2_, methyl-containing compounds and acetate. All of these substrates are stoichiometrically converted to methane. The ability of methanogens to use H_2_ as an electron donor for the restoration of CO_2_ is widespread in natural ecosystems.

A methanogen that metabolizes carbon dioxide is classified as hydrogenotrophic, while those that metabolize the methyl group of acetate are called acetotrophic or aceticlastic. For the past two decades, methanogens isolated from terrestrial habitats have been studied as a model for extraterrestrial life [25,26]. As shown in Mars-modeling experiments, methanogens could exist on a low concentration of molecular hydrogen [27], on a Mars soils stimulant [28,29,30,31,32], survive following desiccation at various pressure values [33,34,35,36], and be sensitive to ultraviolet radiation [37,38].

In May 2008, the Wet Chemistry Laboratory on the board of the 2007 Phoenix Mars Lander performed the first wet chemical analysis of Martian soil. The analysis of three samples, two from the surface and one from a depth of 5 cm, revealed slightly alkaline soil and low levels of salts typically found on Earth [39]. The remaining anions included small concentrations of chloride, bicarbonate, and sulfate. Cations were dominated by Mg^2+^ and Na^+^, with small contributions from K^+^ and Ca^2+^. Unexpected though was the presence of ~0.6% by weight of perchlorate (ClO_4_^−^), most likely as a Ca(ClO_4_)_2_ or Mg(ClO_4_)_2_ phase.

Perchloric acid salts are chaotropic compounds causing the weakening of the hydrogen bonds between water and macromolecules [40,41,42] unlike kosmotropic (stabilizing) salts or substances which may have a comparable effect [42,43]. All chaotropic substances thus far tested have been shown to act on macromolecular systems *in vivo* in studies of diverse microorganisms [44,45,46,47,48]. On the other hand, it has been known for more than 50 years that microorganisms can reduce oxyanions of chlorine such as chlorate (ClO_3_^−^) and perchlorate under anaerobic conditions [49]. The high reduction potential of perchlorate (ClO_4_^−^/Cl^−^*E_o_* = 1.287 V) makes it an ideal electron acceptor for the microbial metabolism. The metabolic capability of perchlorate reduction is widespread throughout the Proteobacteria [50], which has some interesting evolutionary implications in the light of the assumed limited geographical distribution of natural sources of chlorate and perchlorate. However, so far, we know about several representatives of nonmethanogenic *Archaea* that reduces perchlorate [51,52,53], and it is not clear whether these compounds are stressors to methanogens.

In this paper we study the effect of sodium and magnesium perchlorates on growth of permafrost and nonpermafrost methanogens, and present evidence that permafrost hydogenotrophic methanogens are more resistant to the chaotropic agent found in Martian soil.

## 2. Experimental Section

### 2.1. Archaeal Strains

Three strains of methanogenic archaea *Methanobacterium articum* M2^T^ VKM B-2372^T^, *Methanobacterium veterum* MK4^T^ VKM B-2440^T^, and *Methanosarcina* sp. JL01 VKM B-2370 isolated in our laboratory from permafrost of different ages [20,21,22] were tested in the study. *Methanobacterium bryantii* strain M.o.H.^T^ VKM B-1629^T^ and *Methanosarcina mazei* S6^T^ VKM B-1636^T^ from All-Russian collection of microorganisms (VKM) were used for comparative experiments (Table 1).

**Table 1:** Characteristics of methanogenic archaea used in the study.

Methanogens	Substrates for Methanogenesis	Source of Isolation	References
*Methanobacterium bryantii* M.o.H.^T^	H_2_ + CO_2_	Syntrophic association	[54]
*Methanosarcina mazei* S-6^T^	Acetate, methanol, methylamine, trimethylamine, H_2_ + CO_2_	Anaerobic sewage digester	[55]
*Methanobacterium arcticum* M2^T^	H_2_+CO_2_, formate	Holocene permafrost	[22]
*Methanobacterium veterum* MK4^T^	H_2_ + CO_2_, H_2_ + methanol, H_2_ + methylamine	Plieocene permafrost	[21]
*Methanosarcina* sp. JL01	Acetate, methanol, methylamine, trimethylamine	Holocene permafrost	[20]

### 2.2. Media and Cultivation

Media for archaeal strains were prepared according to the Hungate anaerobic technique [56]. Strains *M.arcticum* M2^T^ and *M. bryantii* M.o.H.^T^ were grown in MB medium containing (g·L^−1^) 0.05 g sodium acetate trihydrate, 0.45 g (NH_4_)_2_SO_4_, 0.29 g K_2_HPO_4_, 0.18 g KH_2_PO_4_, 0.12 g MgSO_4_.7H_2_O, 0.06 g CaCl_2_. 2H_2_O, 5.0 g NaCl, 10 mL vitamin solution (medium 141; DSMZ), 10 mL trace element solution (medium 141; DSMZ), 0.001 g resazurin, 0.25 g cysteine hydrochloride hydrate, 0.25 g Na_2_S·9H_2_O; H_2_/CO_2_ (80:20) at 200 kPa; [54] and *M. veterum* strain MK4^T^ was grown in DSMZ medium 506 with the same modification [21]. A basal medium of the following composition was used for cultivation of *M. mazei* strain S6^T^ and *Methanosarcina* sp. JL01 (g·L^−1^): 4.0 g sodium acetate trihydrate, 0.29 K_2_HPO_4_, 0.29 KH_2_PO_4_, 1.0 NaCl, 0.2 MgCl_2_ × 6H_2_O, 1.0 NH_4_Cl, 0.1 CaCl_2_ × 2H_2_O, 0.5 cysteine hydrochloride, plus 5 mL vitamin solution and 10 mL trace elements solution [54].

Methanogens were grown in Hungate tubes or flasks at temperatures that are optimal for the growth of each strain: 37 °C (*M. bryantii* M.o.H.^T^, *M. arcticum* M2^T^ and *M. mazei* S6^T^), 30 °C (*M. veterum* MK4^T^) and 25–28 °C (*Methanosarcina* sp. JL01).Growth was determined by the methane formation in the gas phase, or spectrophotometrically at 600 nm.

### 2.3. Determination of Inhibitory Concentrations

**Figure 1:**
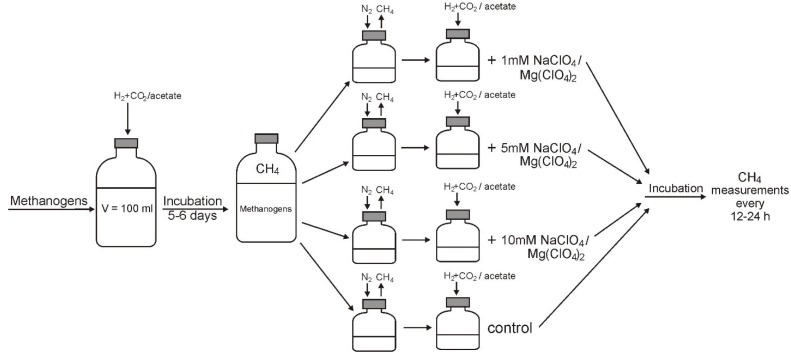
Scheme of the experiment for determining the inhibitory concentrations of perchlorates.

Inhibitory effect of substances on methanogens was assessed by measuring their influence on the rate of methanogenesis from CO_2_ + H_2_ and acetate (Figure 1). Methanogenic strains were grown in 150 mL flasks with 100 mL medium volume under optimal conditions, medium and the substrate for each strain until the optical density (OD_600_) was 0.2 for *Methanobacterium* spp. and 0.3 for *Methanosarcina* spp. It should be noted that both methanosarcina strains grow with a milky white turbidity formation, and do not form large clamps in the used medium.

The scheme of the experiment for determining the inhibitory concentrations of perchlorates can be seen in Figure 1. Grown cultures of methanogens were added to 30-mL glass flasks containing 10 mL of cultivation medium for the respective strain supplemented with 30 mM acetate or with H_2_ + CO_2_ gas phase (80:20, at 200 kPa). After purging methane from the medium with nitrogen, the flasks were incubated anaerobically at temperatures corresponding to each strain.

On the following day, the incubation medium was analyzed for acetate and H_2_ and their concentrations in medium and gas phase was replenished. The perchlorates were added to assay flasks at desired concentrations, except for control flasks free of tested substances. After flushing the flasks with nitrogen, the incubation was resumed. Following two days of incubation, the measurement and replenishment of acetate and hydrogen were repeated as described above. After a 1 h reincubation, the headspaces of all the assay flasks were analyzed for the methane produced during this time period. The methane production assay was continued for two days at 12- to 24-h intervals. The maximal specific rate of acetoclastic and hydrogenotrophic methanogenesis was determined from the slope of the time curve of methane production. Inhibited methanogenic activity was expressed as a percentage of the methane production rate in the control flasks, %ACT. The percentage of inhibition (%I) was defined as %I = 100 − %ACT. The concentrations of compounds that caused 20%, 50%, and 80% inhibition of methanogenesis were referred to as, IC_20_, IC_50,_ and IC_80_, respectively.

### 2.4. Microscopic Examination

**Light microscopy.** Cell morphology was examined under light microscope Opton ICM 405 (Carl Zeiss, Oberkochen, Germany) using the phase-contrast method at a magnification of 100 × 3.5.

**Electron microscopy.** Bacterial cells were pre-fixed with 1.5% (v/v) glutaraldehyde in 50 mM cacodylate buffer (pH 7.2) at 4°C for 1 h, washed thrice in the same buffer, and refixed in a 1% (w/v) solution of OsO_4_in the buffer at 20 °C for 4 h. The sections were dehydrated in a series of alcohol solutions of increasing concentration, embedded in Epon 812 epoxy resin, mounted on a grid, and contrasted in a 3% (w/v) solution of uranyl acetate in 70% (v/v) ethanol and then with lead citrate [57] at 20 °C for 4–5 min.

### 2.5. Analytical Procedures

**Acetic acid and methane** were analyzed using a Pye-Unicam gas chromatograph (United Kingdom). The glass column (1 m × 2 mm ID) for methane assay was packed with Porapak Q, 80–100 mesh (Fluka, Buchs, Switzerland). The column, injector, and flame-ionization detector were kept at 90, 150, and 180 °C, respectively. The carrier gas was nitrogen at a flow rate of 20 mL/min. Acetic acid was assayed on a glass column (2 m × 2 mm ID) packed with a 20 wt% neopentylglycol succinate on Chromosorb W/AW-DMCS, 100–200 mesh (Fluka, Buchs, Switzerland). The pH of samples was reduced to 4.0 by adding ortophosphoric acid. The column temperature was raised from 80 to 175 °C at a rate of 6 °C/min. The injector and detector were kept at 150 and 180 °C, respectively.

**Hydrogen** content was determined by a gas chromatograph Shimadzu 8A equipped with a thermal conductivity detector (TCD) and a stainless steel column packed with a molecular sieve 5Å, 60–80 mesh. The temperatures of the detector and column were maintained at 100 and 60 °C, respectively.

**Perchlorate** concentrations were determined with an IC-2010 ion chromatograph system equipped with a TSKgel SuperIC-Anion HS (Tosoh, Tokyo, Japan). The eluent contained 10 mM NaHCO_3_, 8 mM Na_2_CO_3_, and 30% (v/v) CH_3_CN. The analytical conditions were as follows: flow rate, 1.2 mL/min; column temperature, 40 °C; and injection volume, 30 μL. The limit of quantification was 0.001%. In order to determine perchlorates, 1 mL of methanogenic culture was collected and centrifuged (12,000 rpm) to separate the biomass.

## 3. Results and Discussion

Methane-producing archaea under study were isolated from different habitats (Table 1). Reference strains of hydrogenotrophic archaeon *M. bryantii* M.o.H.^T^ and acetate-consuming archaeon *M. mazei* S-6^T^ are the most abundant methanogenic species widespread in garden soil, sewage sludge, black mud, and feces of herbivorous animals and also isolated from urban solid waste, various sewage and animal waste digesters, swine waste lagoons, and kelp enrichments. Three other methanogenic strains were isolated from Arctic permafrost of different ages. *M. articum* M2^T^ and *Methanosarcina* sp. JL01, hydrogen-and acetate-consuming strain, respectively, were isolated from Holocene permafrost. In contrast, hydrogenotrophic archaeon *M. veterum* MK4^T^ was isolated from ancient permafrost which never thawed during 3 million years. All isolates were psychrotolerant mesophiles most probably responsible for the production of methane in permafrost samples from which they were isolated [20].

### 3.1. Determination of Perchlorates Inhibitory Concentrations

To determine the inhibitory effect of sodium and magnesium perchlorates and a mixture thereof, we used concentrations of up to 10 mM, since the aqueous extract Mars soil found precisely this total amount salt [39] and it is the maximum amount of perchlorates that could be there. Table 2 provides calculations of concentrations of NaClO_4_, Mg (ClO_4_)_2_ and their mixture (1:1), which reduce the methane formation by methanogens by 20, 50, and 80%, respectively. The results shows that, if 2.1 to 9.0 mM Mg(ClO_4_)_2_ was added into the culture medium we observe 20% reduction in the amount of methane by all methanogenic strains. It should be noted that the *M. arcticum* M2^T^ was the most resistant to the perchlorates action. Adding 9.0 and 9.8 mM magnesium and sodium salt, respectively, reduced the methanogenesis *M. bryantii* MoH^T^ by 80%. The joint addition of two perchlorates in all cases increased the inhibition effect. The concentration at which these salts inhibit the cell growth (Table 2) is consistent with their activity as chaotropic stressors [42,48] disordering cellular macromolecules.

**Table 2:** Inhibitory effect of Mg(ClO_4_)_2_ and NaClO_4_ on the growth of permafrost and nonpermafrost methanogens.

Strains	Mg(ClO_4_)_2_, mM			NaClO_4_, mM			Mg(ClO_4_)_2_ + NaClO_4_, mM		
	IC_20_	IC_50_	IC_80_	IC_20_	IC_50_	IC_80_	IC_20_	IC_50_	IC_80_
*M. bryantii* M.o.H^T^	3.5	6.2	9.0	2.8	6.0	9.8	2.2	4.0	8.1
*M. arcticum* M2^T^	9.0	>10.0	>10.0	>10.0	>10.0	>10.0	>10.0	>10.0	>10.0
*M. veterum* MK4^T^	2.6	6.6	>10.0	4.1	8.4	>10.0	2.5	5.6	9.0
*M. mazei* S-6^T^	5.0	9.2	>10.0	7.8	>10.0	>10.0	4.8	>10.0	>10.0
*Methanosarcina* sp. JL01	2.1	5.2	>10.0	3.9	9.7	>10.0	1.8	4.8	>10.0

### 3.2. Methane Generation in Perchlorate-Supplemented Medium

**Figure 2:**
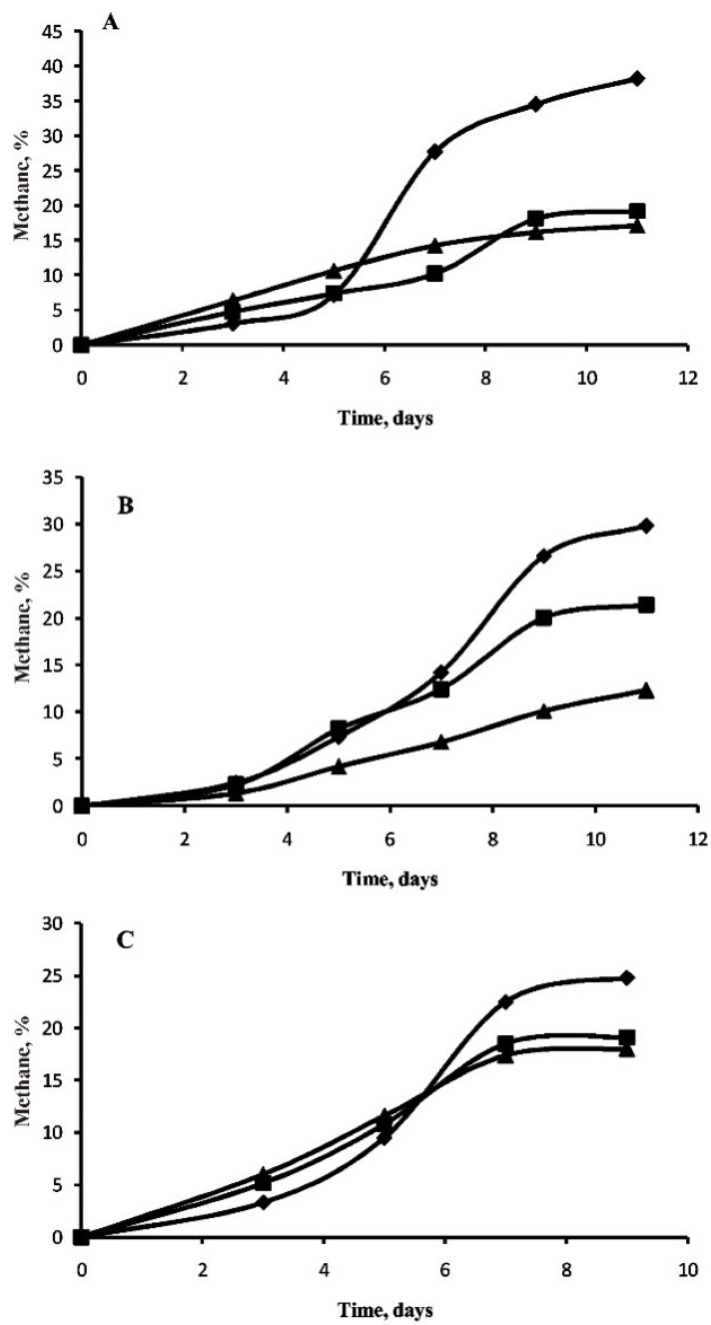
Methane formation by hydrogenotrophic strains *M. bryantii* M.o.H.^T^ (**A**), *M. veterum* MK4^T^ (**B**) and *M. arcticum* M2^T^ (**C**) with NaClO_4_ (square), Mg(ClO_4_)_2_ (triangle) and without perchlorates (rhombus).

All studied microorganisms were used to test the possible growth and methanogenesis in media supplemented with two perchlorates salts which were added in final concentration of 5 mM. Growth of methanogens is characterized by an extremely low biomass accumulation and usually it is judged by the formation of methane in the gas phase and the amount of methane formed by microorganisms being proportional to the biomass produced [58]. Investigation of the influence of sodium perchlorate and magnesium on methanogenesis by hydrogen-consuming methanogens confirmed the stability of methanogens isolated from permafrost in the perchlorate-supplemented media (Figure 2A–C). The growth of *M. bryantii* is inhibited to a much greater extent than the growth of *M. veterum* MK4^T^ and *M. arcticum* M2^T^. The growth of strain *M. arcticum* M2^T^ was characterized by a greater rate in the presence of perchlorates (Figure 2C); however, there was 20% less methane formed by the strain than in the control that does not contain perchlorates.

**Figure 3:**
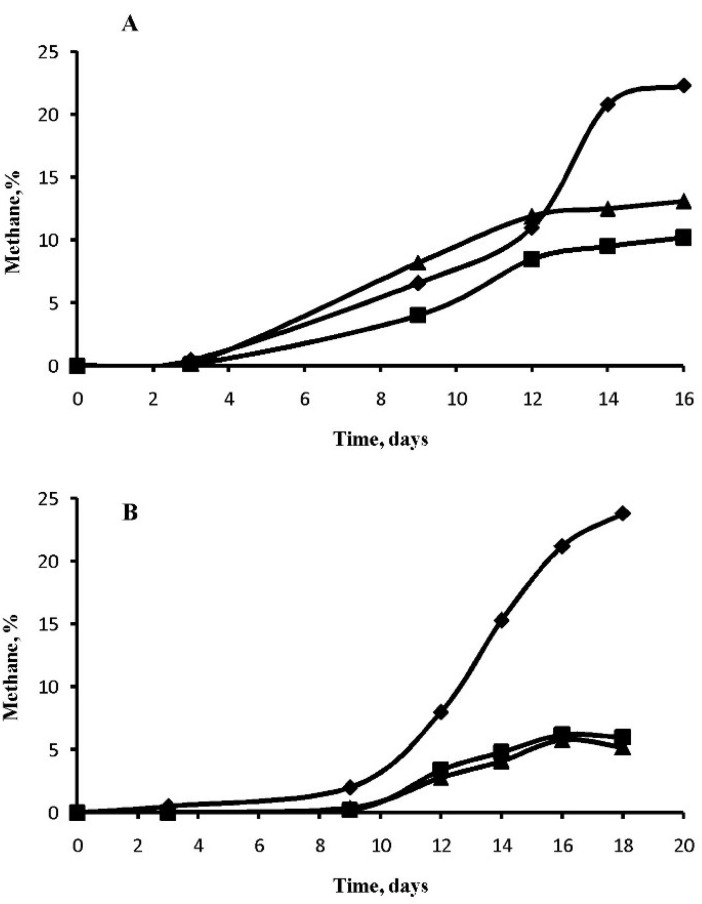
Methane formation by acetoclastic strains *M. mazei* S-6^T^ (**A**) and *Methanosarcina* sp. JL01 (**B**) with NaClO_4_ (square), Mg(ClO_4_)_2_ (triangle) and without perchlorates (rhombus).

Study of the NaClO_4_ and Mg(ClO_4_)_2_ effect on methanogenesis from acetate by *Methanosarcina* spp. showed, that in this case, perchlorates slow down the process more heavily, and additionally also result in the elongation of the lag phase to nine days for strain JL01 (Figure 3). The exception was *M. mazei* S-6^T^ growth in the presence of sodium perchlorate, when the growth rate exceeded the growth rate of methanosarcina in the medium with no perchlorates.

It is known that a chaotrope-induced stress can then trigger oxidative stress [42,44], in particular with oxygen, which in this case may be formed from perchlorate anion [50]. Salt tolerance is expected to be a significant requirement for survival on Mars, and hypersaline environments on Earth, especially in permanently cold regions, are primarily discussed as analogs for Martian brines [10]. These include cryopegs within permafrost [59], brine veins within glacial ice [60], or evaporation ponds in the dry regions of Antarctica [61] and deep-sea hypersaline brine lakes [45]. A recent study of microbiology within the seawater: brine interface at deep-sea hypersaline brine Lake Kryos reports recovery of mRNA at higher levels of MgCl2 (2.27–3.03 M) [62]. It is halophilic species that are most likely to be able to grow under these conditions, and indeed at subzero temperatures, because ion concentrations are increased [63,64].

Earlier the study of microorganisms from Arctic cryopeg showed that the majority of their inhabitants are not halophilic but halotolerant representatives of *Eukarya*, *Bacteria*, and *Archaea* [59]. We have also obtained data that halotolerance of nonhalophilic psychrophilic and psychrotrophic bacteria isolated from cryopeg increases when the cultivation temperature decreases [65]. In the meantime, there is evidence that chaotropic substances (including salts/ions) can actually increase metabolic activity and multiplication of microbes at very low temperatures because they enable the cell to retain biologically permissive membrane fluidity [42,64].

The methanogens used in the study are not halophiles, but all the strains are tolerant of the NaCl concentration up to 0.3 M in the medium [22]. But, unlike *M. bryantii* M.o.H.^T^ and *M. veterum* MK4^T^, which grow best in fresh medium, *M. arcticum* M2^T^ showed the highest growth rate at a NaCl concentration of 0.1 M. Perhaps this difference in physiology of the studied methanogens explains why the response to stressors such as perchlorates differs. Further studies will clarify whether perchlorate- and other chaotropic salts are stressors or they may actually promote or enable growth at some temperatures [10].

Our study results of the methanogens growth in the presence of strong oxidants have put a new question forward: can methanogens use the anion ClO_4_^−^ as an electron acceptor in the oxidation of methane which they were produced? It was in as early as 1979 that methanogenic archaea of *Methanobacterium*, *Methanospirillum*, and *Methanosarcina* genera were found to form and oxidize methane at the same time [66]. As compared to the quantity of methane formed, the amount of methane simultaneously oxidized varied between 0.3% and 0.001%, depending on the strain used.

It is now known that the anaerobic oxidation of methane (AOM) is a significant process in the global carbon cycle and an important sink of methane on Earth. As shown by recent studies, this process is carried out by anaerobic methanotrophic archaea (ANME). Initially, ANME were defined as a separate branch within Euryarchaeota, capable of receiving energy from the anaerobic methane oxidation while using sulfate as a terminal electron acceptor [67]. However, recently it was reported that as a terminal electron acceptor, these microorganisms could utilize nitrate [68] and compounds of iron and manganese [69], not only in marine, but also in freshwater habitats. Using molecular detection tools it was found that methane-producing archaea of *Methanosarcina* genus are the main group of methanogens found in permafrost grounds [70,71], and many of the detected ANMEs are genetically close to the *Methanosarcinales* order. Thus, the assumption about the use perchlorates as acceptors instead of sulfates in the anaerobic oxidation of methane is not unreasonable, given the recently appeared first publication describing the use of magnesium perchlorate by *Archaeglobus* sp. as an electron acceptor instead of sulfate [51]. Due to these speculations, we measured how perchlorates concentration changed after nine days of the *Methanobacterium* spp. cultivation and 16 days of *Methanosarcina* spp. cultivation. The medium without the addition of methanogen inoculum incubated the same time was served as an abiotic control. It should be noted that in the case of *Methanosarcina* spp. the decrease of the perchlorates content was less than 10% of the amount initially added in all cases, including control.

**Figure 4:**
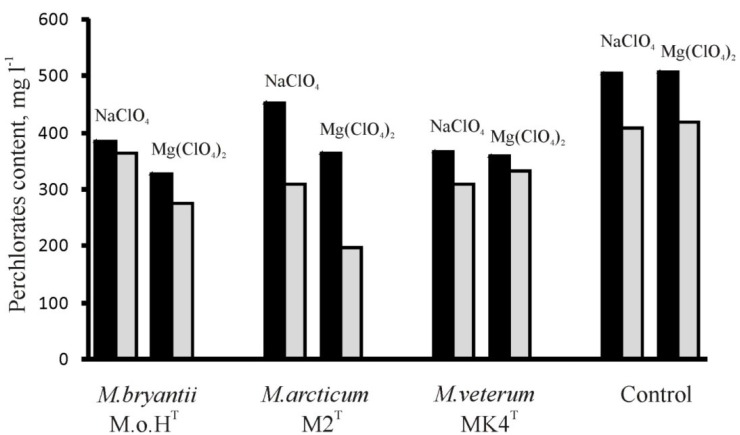
The perchlorates content during the cultivation of hydrogen-consuming methanogens. Black bars—perchlorate concentration in the medium at the initial point; gray bars—the perchlorate concentration after nine days of growth. The control was MB medium supplemented with perchlorate salts.

Another pattern (Figure 4) was observed in determining the content perchlorates before and after cultivation of *Methanobacterium* spp. When strains *M. bryantii* M.o.H.^T^ and *M. veterum* MK4^T^ were cultured, the change in NaClO_4_ (5.7%–16.1%, respectively) and Mg(ClO_4_)_2_ (16.0%–7.2%, respectively) content did not exceed the reduction of the perchlorates concentration in the control (19.0% and 17.6%, respectively). In contrast, the content of perchlorates in the culture medium *M. arcticum* M2^T^ decreased by 31.8% and 45.6%, respectively. Therefore, the decrease of the perchlorates concentration during the strain growth suggests the possible use of the perchlorate anion as an electron acceptor for the oxidation of methane. This remarkable result, of course, requires experimental confirmation, but also offers opportunities to explore new unusual ways of obtaining energy for the methanogens, including in extraterrestrial environments.

### 3.3. Effect of Perchlorate on Morphology of M. arcticum M2^T^ Cells

**Figure 5:**
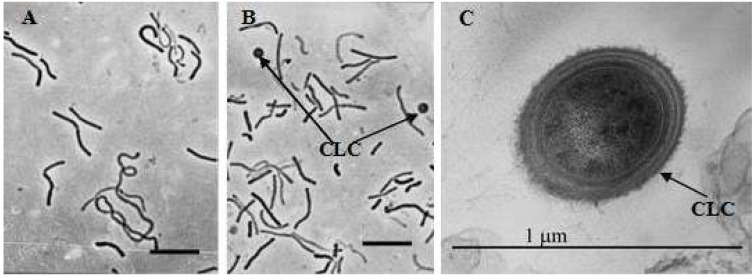
Micrographs of strain *M. arcticum* M2^T^ cells without perchlorate (**A**) and with Mg(ClO_4_)_2_ in the medium (**B**,**C**): **A**,**B**—phase contrast, bar 10 µm; **C**—ultrathin section. Abbreviations: CLC—cyst-likes cells.

In order to understand the reasons for the high resistance of the strain *M. arcticum* M2^T^ to perchlorates, we carried out a microscopic study and examined the morphology of cells grown in the medium supplemented with Mg(ClO_4_)_2_ as a stronger oxidizer. Typically, cells of the strain were non-motile, slightly curved rods (Figure 5A), 0.45–0.50 µm wide and 3.0–6.0 µmlong, often forming chains and filaments more than 30 µm long. Previously we have shown that during long-term storage, strain *M. arcticum* M2^T^ formed cyst-like coccoid cells [22]. However, in the presence of perchlorate the formation of cyst-like cells began during the logarithmic growth phase. The cytoplasm of cyst-like cells was denser, and differentiated surface layers were observed (Figure 5C). The ability to form such morphotypes differentiates *M. arcticum* M2^T^ from other methanogens used in the study and are probably makes this strain resistant to perchlorates.

If life existed during the early stages of Martian development, the remnants of primitive forms may be found within frozen material that protects them against unfavorable conditions [10]. Here we consider terrestrial permafrost analogues as a bridge to possible Martian life forms and shallow subsurface habitats where the probability of finding life is the highest. Since there is a place for water, the requisite condition for life, even in solid phase, these analog models are more or less realistic. Permafrost on Earth and Mars vary in age, from a few million years found on the Earth to a few billion years on Mars; such a difference in time scale would have a significant impact on the possibility of preserving life on Mars because the number and biodiversity of microorganisms decrease with increasing permafrost age. This is why the known longevity of life forms preserved within the permafrost can only work as an approximate analogue for Mars.

However, because at subzero temperatures the rate of biochemical reactions and biological process becomes extremely low, microbiological methods are not sufficiently sensitive to detect cell activity. Using ^14^C-labeled substrates, the possibility of methane formation within permafrost was experimentally demonstrated at subzero temperatures as low as −16.5 °C [20,72]. The experiments with radiolabeled substrates showed that methanogenic archaea not only preserve their viability in the permafrost, but also are able to realize metabolic reactions at subzero temperatures, perhaps it involves chaotropic agents. At these temperatures, autotrophic microorganisms from both the relatively young (2920 years) Holocene permafrost (*M. arcticum* M2^T^ and *Methanosarcina* sp. JL01) and the old (~3 million years) Plieocene deposits (*M.veterum* MK4^T^) a metabolically active [73].

## 4. Conclusions

Furthermore, as shown in the studies presented here, methane-producing microorganisms isolated from a permafrost were resistant to oxidizing agents such as a perchlorates, and strain M2^T^ *M. arcticum*, probably can use perchlorate as an electron acceptor in the anaerobic methane oxidation.

Methane-producing microorganisms from the *Archaea* domain of life have evolved features distinct from all other life forms, particularly cofactors and several enzymes essential for the pathways to produce CH_4_ from reduction of the methyl group of acetate or CO_2_. Although understanding of these pathways has derived primarily with methanogens isolated from organic rich halophilic and nonhalophilic surface environments, features of the pathways also apply to organic-poor environments [24]. Earth’s subzero subsurface environments are the best approximation of environments on Mars, which is most likely to harbor methanogens; thus, a biochemical understanding of these pathways are expected to provide a basis for designing experiments to detect autotrophic methane-producing life forms on Mars.
